# Skill and Strategy: How Managerial Ability Drives Working Capital Efficiency

**DOI:** 10.12688/f1000research.162551.1

**Published:** 2025-03-28

**Authors:** Prasenjit Roy, Sagar Bhatt, Rakesh Dani, Premendra Kumar Singh, Asokan Vasudevan, Elangbam Nixon Singh

**Affiliations:** 1 Department of Commerce, Tezpur University, Napaam, Assam, 784028, India; 2 Graphic Era Deemed to be University, Dehradun, Uttarakhand, 248002, India; 3 Centre for Distance and Online Education, Sharda University, Greater Noida, Uttar Pradesh, 201308, India; 4 Faculty of Business and Communications, INTI International University, Nilai, 71800, Malaysia; 5 Department of Management, Mizoram University, Aizawl, Mizoram, 796004, India

**Keywords:** Managerial Ability, Working Capital Management Efficiency, Data Envelopment Analysis, Generalized Estimating Equations (GEE), Financial, Economic

## Abstract

**Background:**

The study investigates the impact of Managerial Ability (MA) on Working Capital Management Efficiency (WCME), focusing on how skilled management influences working capital practices. It also explores the variation in this relationship across firms with different characteristics such as profitability and market valuations within India’s economic landscape.

**Methods:**

Using a balanced panel dataset of 150 listed companies from the National Stock Exchange (NSE) of India for the period 2014–2023, the study employs Data Envelopment Analysis (DEA). WCME is assessed as a multidimensional efficiency metric incorporating inventory, accounts receivable, accounts payable, cost of goods sold , net revenue, and net income. MA is estimated using a two-step DEA-based approach, separating managerial ability from firm-specific characteristics. Generalized Estimating Equations (GEE) regression models are applied to examine the individual and interactive effects of MA and Tobin’s Q (TBQ) on WCME along with other important variables.

**Results:**

The results demonstrate that MA significantly enhances WCME, particularly in firms with lower TBQ. The analysis also reveals that skilled managerial characteristics amplifies the efficiency of working capital management, aligning with the Upper Echelon Theory's (UET) claim that managerial attributes play a critical role in organizational efficiency.

**Conclusion:**

The study demonstrates managerial ability has a strong influence on corporate working capital policies. This advocates prevalence of certain fundamental concepts such as viz. Upper Echelon Theory (UET), Resource-Based View (RBV), and Agency Theory (AT). From managerial point of view, it is suggested to adopt strategic approaches in practicing WCME that will enhance firm’s competitive advantage and long-term sustainability.

## 1. Introduction

Resource management is crucial for firms to achieve operational and financial objectives. Managers optimise allocating overall resources and short-term capital to drive profitability and long-term sustainability. Managerial Ability (MA) and Working Capital Management (WCM) plays a strong role towards impacting corporate performance ( Banerjee & Deb, 2024). MA is the term which is used to reflect a manager’s skill, knowledge, and decision-making capacity. This has a direct effect on liquidity management, operational efficiency, and long-term growth of a firm. It is closely linked to efficient working capital management practices, without which a firm's overall financial stability gets affected ( Ahsan & Mostafa, 2017; Andreou et al., 2017). WCM in targeted towards maintaining liquidity and operational requirement of an organisation. This is done through a strategic approach of handling current assets and liabilities. It is believed that capable managers have better strategic insight and market awareness that helps to optimize cash flow, manage receivables, and control payables ( Abdullah et al., 2022; Deloof, 2003). Thus, it would not be inaccurate to say that a high MA may boost WCM, and help firms meet their financial obligations. It acts as a foundation for the firms’ long-term prosperity and growth.

MA and WCM has a complex and dynamic relationship due to various factors. This is because both short-term goals and long-term success is highly dependent upon strategic decision-making. High-ability managers may prioritize future growth over immediate working capital efficiency, potentially affecting short-term liquidity but fostering long-term profitability ( Banerjee & Deb, 2023, 2024; Ujah et al., 2020). They predict market shifts, strengthen supplier relationships, and navigate regulatory challenges; crucial for optimizing WCM and sustaining a competitive edge ( Demerjian et al., 2013; Ulaga & Eggert, 2006). Balancing short-term efficiency with long-term profitability shapes financial stability and growth.

There are limited studies on the linkage between MA and WCE. While there are numerous studies which examine their independent effects on corporate performance ( Ahsan & Mostafa, 2017; Banos-Caballero et al., 2012; Cornaggia et al., 2017; Deloof, 2003), however, there are only few which explores how MA directly influences WCM ( Banerjee & Deb, 2023, 2024; Ujah et al., 2020). The findings are inconclusive in confirming the exact nature of association between MA and WCM. This creates a research lacuna for emerging markets like India. It is important due to the fact that India has a unique institutional and regulatory environment which shapes financial strategies of the firms more profoundly against global comparison. It poses a distinctive challenge for the firms to balance short-term liquidity and long-term growth at the same timemaking MA a catalyst in WCM decisions. Hence, an understanding of how MA impacts WCM in such settings would offer a valuable insight for the firms. The research is directed towards understanding the effect of MA on WCME within largest listed firms of India. The findings will offer understandings into managerial skill and its role in refining working capital policies. The research is structured in the following manner where Section 2 highlights literature review and hypotheses. Section 3 presents the research methodology. The findings and implications are presented in Section 4 and lastly conclusion in Section 5.

## 2. Literature review

### 2.1 Conceptual context

*2.1.1 Working capital management*

A corporation's financial health is highly dependent on how both long and short-term resources are managed. WCM provides a strategic approach for managing short-term assets and liabilities ( Beaumont Smith & Begemann, 1997). It has been argued that WCM efficiency is essential in enabling businesses to meet short term obligations alongside increasing profitability ( Deloof, 2003). The trade-off theory related to working capital suggests that there must be an equilibrium between the advantage and disadvantages of managing liquid assets. For instance, an excessive cash, receivables, and inventory may prohibit firms to reach profitable position and lead to opportunity costs ( Banos-Caballero et al., 2012; Deloof, 2003; Nazir & Afza, 2009). On the other hand, working capital deficiencies leads to rigidity in the liquidity position and disrupts the day to day activity of the firms ( Aktas et al., 2015; Baños-Caballero et al., 2014). Firm value is only possible with the effective management of current assets (CA) and current liabilities (CL) which are elements of working capital ( Banos-Caballero et al., 2012). This would ensure steady cash flows, operational effectiveness, and better firm performance. It aligns with the essence of Agency Theory (AT), in such a manner where better WCME alienates the possibility of conflicts among the shareholders and managers. Apparently, certain managers may hold excess working capital funds as a cushion against liquidity crisis. It will most likely create a conflict among the shareholders as they will perceive this act as resource misallocation thereby reducing the firm value ( Jensen & Meckling, 1976).

A foundational aspect of working capital is to have an ideal WCM strategy. There are two important approaches in this context. They are known as Working Capital Investment Policy (WCIP) and Working Capital Financing Policy (WCFP) ( Addin Al-Mawsheki, 2022; Nazir & Afza, 2009). The WCIP considers magnitude of CA with regard to Total Assets (CATA). Whereas, WCFP emphasises on management of CA through a mix of CL, equity, or long-term debt (CLTA) ( Addin Al-Mawsheki, 2022; Nazir & Afza, 2009). The approaches are aligned in two manner either conservative or aggressive, based on the firms’ capability to withstand risks and strategic positioning ( Nabi et al., 2016). High CATA ratio implies a conservative approach where the firm prioritises liquidity and maximum threshold for operational disruptions ( Honda & Uesugi, 2022). This enhances investment towards yielding customer satisfaction and increased sales ( Pant et al., 2023). The aggressive WCM strategy on the hindsight focuses on external finance for managing the short term obligations ( Nazir & Afza, 2009). This approach creates more risk for the firms but if managed effectively can bear fruitful results ( Baños-Caballero et al., 2014). The route to effective working capital strategy has immense repercussions for a firm’s financial position, risk and market value ( Ahmad et al., 2022; Honda & Uesugi, 2022; Nabi et al., 2016; Pant et al., 2023; Roy et al., 2025). An effective WCME ensures that firms reduce operational expenditure and at the same time have adequate liquid assets to meet the short-term obligations. This has been indicated to be essential for organisational growth and sustainable business performance ( Akgün & Memiş Karataş, 2021; Aktas et al., 2015; Deloof, 2003; Nabi et al., 2016; Ren et al., 2019).

Assessment of WCM involves either a static or an operating cycle approach. The static approach is based on financial statements particularly the balance sheet. An examination of CL gives us Gross Working Capital, whereas the difference between CA and CL gives Net Working Capital (NWC) ( Brigham & Ehrhardt, 2015). The operating cycle approach considers Cash Conversion Cycle (CCC) as avital element. The focus, in this is on the duration needed for cash transformation starting from inventory management, sales and then to cash. For this, metrics such as accounts receivable, accounts payable, inventory holding period, purchase and sales activities act as important measurements ( Deloof, 2003; Nabi et al., 2016; Richards & Laughlin, 1980). These measurements collectively act as important factors in designing effective WCM strategies where the various angles of corporate decisions such as liquidity, operational efficiency and profitability are maintained sustainably.

*2.1.2 Managerial ability*

Corporate performance is influenced by various factors among which MA plays decisive role ( Baik et al., 2020). The concept of MA integrates a range of characteristics of managers such as competency in decision-making, management of resources, strategy formulation and implementation that drives short term profitability and long term sustainability ( Ahsan & Mostafa, 2017; Baik et al., 2020). The Resource-Based View (RBV) lays down an important framework within this where organisation’s success is highly dependent on management of both tangible and intangible resources ( Barney, 1991). It augments firms towards competitive advantage and makes them leader in their respective sectors. High MA thus appears to be a resource, which is unique, rare, valuable, and inimitable in nature. It is necessary for the firms to achieve a desired operational efficiency ( Banerjee & Deb, 2024). Skilled managers are differentiated by their capacity to allocate resources effectively and convert their decisions into high performance outputs ( Demerjian et al., 2013).

Additionally, the Upper Echelons Theory (UET) also suggests that Top Executives in any organisation have significant influence towards attainment of objectives. The linkage to MA can be made with managers who have higher ability to perceive and evaluate market dynamics in such a manner that it transpires growth effectively ( Ahsan & Mostafa, 2017; Hambrick & Mason, 1984). Adaptability of the managers to business environment, productivity and resiliency becomes inherent within MA. This is explained through the Dynamic Capability Framework (DCF) ( Baik et al., 2020; Teece et al., 1997). Also, connecting the idea to Agency Theory, it is imperative that high MA induces managers to incline towards the shareholder’s objective, reduce agency conflicts and maintain transparency ( Cornaggia et al., 2017; Jensen & Meckling, 1976).

MA has been measured in a varied manner throughout numerous studies. However, it has been inconclusive as to which measure gives the better indication. This is because the gamut of managerial decision-making is wide and touches every corner of an enterprise. There have been notable attempts to quantify MA, however, it remains to be complex and challenging ( Bertrand & Schoar, 2003; Demerjian et al., 2012). Previously studies have used various proxies of MA based on manager’s education, managerial tenure and achievements ( Bertrand & Schoar, 2003; Fee & Hadlock, 2003). However, there were limitations in these proxies that paved way to the development of more robust method. Demerjian et al. (2012) suggested a reasonable measurement process of MA in their seminal work. It consisted of a two staged approach where firstly corporate efficiency needs to be calculated using Data Envelopment Analysis (DEA) and a first stage regression. After which, in the second step MA is computed based on residual from the regression result. The residual set aparts impact of firm-specific characteristics from manager-specific factors. For the DEA computation seven inputs are considered. The inputs are viz. Plant, and Equipment, Net Property, operating lease, cost of goods sold, general, selling and administrative expenses, goodwill, R&D expense, and other intangible assets alongside sales as the output variable ( Demerjian et al., 2012). Since its introduction, the computational method has been largely adopted in various fields of studies for computing MA ( Andreou et al., 2017; Banerjee & Deb, 2023, 2024; Cornaggia et al., 2017; Demerjian et al., 2013; García-Meca & García-Sánchez, 2018; Ujah et al., 2020).

*2.1.3 Managerial ability and working capital management*

The connection of MA, WCM with corporate performance can be understood from the various theories mentioned in the previous section. As highlighted, the UET lays down the idea that senior executives are instrumental in corporate outcomes ( Hambrick & Mason, 1984). High skilled managers show better competence and experience towards resource utilisation and decision making enabling them to implement superior WCM. On the other hand, less capable managers may engage in individual opportunistic behaviour prioritising personal goal and aims. This gives rise to agency costs and create a distaste among the shareholders. Better managers are more likely to act ideally for their respective firms. In this line, Agency Theory as suggested by ( Jensen & Meckling, 1976) explains how MA impacts WCM by addressing the potential strife between managers and shareholders’ interests. Skilled managers ease risks related to overinvestment or underinvestment, as they align decisions with shareholder value maximization. Likewise, skilled managers are better able to traverse the sea of organisational complexities and consider a stakeholder perspective towards implementing a well-balanced WCM policy as suggested through the Behavioral Theory of the Firm ( Cyert & March, 1963). From, adaptability angle, those managers who reconfigure working capital strategies corresponding to the variations in the environment are believed to be more skilled. Many such examples of corporations could be pointed out where economic crisis and uncertainty in recent times challenged the liquidity position of firms ( Pant et al., 2023; Roy et al., 2025). However, skilled managers through their adaptable strategy ensure that their firms ensure liquidity, cash flows and operational efficiency. This is particularly characterised by the Dynamic Capability Theory ( Baik et al., 2020; Teece et al., 1997). High MA managers are also expected to manage their short-term fund requirements through internal sources of finance. The Pecking order theory put forwards this concept ( Myers & Majluf, 1984), where this helps to lower the cost of capital and prevent sending a negative signal to the market. They also excel in managing an ideal operating cycle, catering to the needs of the customer as well as the organisation ( Banerjee & Deb, 2023; Roy et al., 2025). Through an efficient management of inventory levels, the transportation costs gets reduced and stock outs are avoided. Skilled managers negotiate beneficial terms with suppliers, ensures efficient cash outflows and strong supply chain relationships helping in efficient receivable management ( Roy et al., 2025). There is a considerable effect of MA on WCM which further impacts corporate performance ( Ujah et al., 2020).

The number of earlier studies in this regard is bleak. Specifically, there are only three studies that focus on this area ( Banerjee & Deb, 2023, 2024; Ujah et al., 2020). Nevertheless, they offer conflicting evidence about the link between MA and WCM. Ujah et al. (2020), reported managerial inefficiency towards maintaining WCME. Their study shows that talented managers were less efficient at managing their firms’ operational needs. They theorized that this inefficiency may stem from talented managers prioritizing long-term projects over short-term operational needs. They corroborate that managers with higher MA tend to take extended duration for converting inventory into sales but are more efficient at extending their payables. They also suggest that operational efficiency might not be the primary driver of increased firm value which is often attributed to talented managers. Another study ( Banerjee & Deb, 2023, 2024) which looks at the connection between corporate performance, capital expenditure (CAPEX), WCM, and MA reveal an inverse relationship between the two. The findings suggests that more able managers show greater WCM efficiency, positively affecting corporate performance . They found that this relationship was non-linear i.e. managers with lower MA scores were less efficient in managing working capital, while managers with higher scores displayed increased efficiency, which grew at a decreasing rate beyond a certain threshold. Banerjee and Deb (2023, 2024) offer arguments to explain the discrepancy between their findings and those of Ujah et al. (2020). They also contradict the inferences of Ujah et al. (2020), which suggest that talented managers, influenced by compensation structures prioritize long-term goals, and tend to overlook short-term operational aspects like WCM. As an alternative, Banerjee and Deb (2023) argue that managers strive to balance WCM and CAPEX in order to increase performance. They report association of higher MA with both higher CAPEX and efficient WCM. It is implied that managers with greater capability look to generate internal funds through WCM. This further streamlines towards helping greater investments in CAPEX. The study highlights the importance of efficient WCM and strategic CAPEX decisions in the Logistics and Transportation industry. Existing research offers contrasting view on the nature of the relationship between MA and WCM. Thus, we see an inconsistency in the findings. This highlights the need for further investigation towards developing a clear understanding of the MA-WCM relationship. Therefore, using the information, the following hypothesis is formed:

H1: Higher MA is expected to be positively associated with greater WCME among Indian firms.

## 3. Methods

### 3.1 Data source and origin

The CMIE Prowess database was used to collect the required data. Spanning across ten years, from 2014 to 2023 the study considers 150 listed corporations from the National Stock Exchange (NSE). The dataset is further refined based on the market capitalization for 2023 and data availability. The selection criteria also considers removal of corporations from specific industries, such as banks, financial institutions, utilities, and ICT, because of their distinct WCM characteristics ( Roy et al., 2025). Corporations having negative net sales or total assets have been excluded to prevent conflicting outcomes ( Deloof, 2003). Furthermore, corporations with partial or missing data are also regarded as an omitting criterion to preserve correctness and consistency. Over a ten-year period, 1500 balanced panel observations from 150 enterprises make up the final sample. As recommended ( Cooper et al., 2007; Sarkis, 2007), the quantity of observations justifies the application of the DEA technique. Across the population, the National Industry Classification (NIC) which provides Industry and Sector data of Indian companies have been used to calculate MA ( Demerjian et al., 2012).

### 3.2 Variables

The variables for the study are presented in Table 1. The WCME is treated as the dependent variable to evaluate its relationship with other variables. It is measured through DEA. The Accounts Payable (AP), Inventory (INV), Accounts Receivable (AR), and Cost of Goods Sold (COGS) were considered as inputs while outputs were Net Revenue (NR) and Net Income (NI). Using WCME ( Habib & Dalwai, 2024), as a dependent variable offers a broader perspective on efficiency than traditional measures such as CCC. While CCC measures only the conversion time, WCME incorporates a broader set of inputs (inventory, COGS, AR, AP) and outputs (net revenue, net income), providing a multidimensional aspect. A higher WCME score shows better efficiency.

**Table 1.** Variables for the study.

TOOLS	INDICATOR	VARIABLES	FORMULA	NATURE
DEA BASED	Managerial Ability (MA)	Net PPE Cost of goods sold Selling, general and administrative expense Operating lease R&D expense Goodwill Other tangible assets	*----------*	INPUTS
		Net Sales	*Net Sales book value at the end of year ‘t’ for a DMU.*	OUTPUT
	Working capital Management Efficiency (WCME)	Inventory (INV)	*Inventory book value at the end of year ‘t’ for a DMU.*	INPUT
		Cost of goods sold (COGS)	*Cost of goods sold book value of year ‘t’ for a DMU.*	INPUT
		Accounts receivables (AR)	*Accounts receivables book value at the end of year ‘t’ for a DMU.*	INPUT
		Accounts payable (AP)	*Accounts payable book value at the end of year ‘t’ for a DMU.*	INPUT
		Net Revenue (NR)	*Net Revenue book value at the end of year ‘t’ for a DMU.*	OUTPUT
		Net Income (NI)	*Net Income book value at the end of year ‘t’ for a DMU.*	OUTPUT
GEE REGRESSION BASED	Working Capital Management Efficiency (WCME)	WCME	Working Capital Management Efficiency (WCME) Score derived from DEA (MPI)	DEPENDENT
	Managerial Ability (MA) Score	MA	Based on Residuals of Regression on Firm Efficiency	INDEPENDENT
	Firm Performance	Return on Assets (ROA)	$\frac{\text{EBITDA}}{\text{Total Assets}}$	CONTROL
		Tobin's Q (TBQ)	$\frac{\text{Market Value of Shares}}{\text{Book Value of Shares}}$	CONTROL
	Interaction Term (MA*TBQ)	MA*TBQ	*Interaction term of MA and TBQ*	CONTROL
	Firm Leverage	LEV	*Debt to Equity*	CONTROL
	Firm Size	SIZE	*Natural logarithmic transformation of total assets*	CONTROL
	Sales Growth	SGR	*Current year sales-previous year sales) /previous year sales*	CONTROL
	Business Affiliation	*DBA*	*Dummy Variable: 1 = Affiliated Firms, 0 = Non- Affiliated Firms*	CONTROL
	Ownership Concentration	*DOC*	*Dummy Variable: 1 = Ownership more than 50 p.c., 0 = Less than 50 p.c.*	CONTROL

Source: Authors Compilation.

MA is used as an independent variable. This measure captures how variations in managerial skill impact performance metrics, operational efficiency, and strategic decision-making across firms. MA is measured using the two-step approach ( Demerjian et al., 2012). First, corporate productivity is estimated via DEA, which compares sales revenue (output) against inputs such as operating expenses, goodwill, R&D, COGS, net PP&E, and intangibles. DEA sets up an efficiency frontier to benchmark the corporations. Secondly, MA is derived as a residual from the first step regression which controls for corporation specific characteristics, isolating managerial efficiency. This method highlights how effectively managers use resources to enhance revenue, providing a robust measure of their impact on firm performance ( Demerjian et al., 2012).

The model also incorporates control variables which are wellestablished determinants of working capital. Specifically, control are applied for return on assets (ROA), Tobin’s Q (TBQ) which serve as measures for financial performance ( Banerjee & Deb, 2024; Ujah et al., 2020), leverage (LEV) ( Ahmad et al., 2022; Banerjee & Deb, 2024; Myers & Majluf, 1984; Ujah et al., 2020), firm size (SIZE) ( Berger & Ofek, 1995) and SGR for sales growth ( Ahmad et al., 2022; Altaf & Ahmad, 2019; Altaf & Shah, 2018; Baños-Caballero et al., 2010, 2014; Nazir & Afza, 2009; Ren et al., 2019). Additionally, dummy variables are introduced to account for business affiliation (DBA) ( Khanna & Yafeh, 2007) and ownership concentration (DOC) ( Shleifer & Vishny, 1997). These controls help isolate the effect of MA on WCME. To assess how the relationship between MA and WCME varies with market value, an interaction term, MA*TBQ, is introduced in the regression model ( Ujah et al., 2020). TBQ is included as a dummy variable, coded as 1 for firms with TBQ>1 and 0 otherwise. We decide from this interaction if the impact of MA on WCME varies for enterprises with high or low market value. A significant interaction term would suggest that the impact of managerial ability on WCME is contingent upon the firm’s growth potential and perceived market value. The second interaction term employs a quadrant-based classification methodology ( Lang et al., 1991), and a modified version of it ( Ujah et al., 2020) as explained below where MA and TBQ are considered as specification criteria. Here, the median values of TBQ and MA are used to categorise the firms into four quadrants or groups. They are as follows:

High TBQ and High MA (HQHM): Firms with superior market valuation coupled with highly skilled management.

High TBQ and Low MA (HQLM): Firms with high market valuation but less skilled management.

Low TBQ and High MA (LQHM): Firms with low market valuation but highly skilled management.

Low TBQ and Low MA (LQLM): Firms with poor market valuation and less skilled management.

### 3.3 Models

*3.3.1 DEA Models*

The application of DEA was found suitable to compute WCME and MA following ( Demerjian et al., 2012; Habib & Dalwai, 2024) respectively. A non-parametric procedure, DEA assesses decision-making units’ (DMUs) efficiency without relying on predefined weights or data distributions ( Cooper et al., 2007; Mourad & Luther, 2022). MA is estimated using a two-step process: first, DEA computes corporate efficiency annually for each firm; second, residuals from a regression model isolate MA by excluding firm-specific characteristics. The Malmquist Productivity Index (MPI) complements DEA to track productivity changes in WCME over time. Efficiency scores between 0 and 1 are obtained by applying the Banker, Charnes, and Cooper (BCC) model, which takes variable returns to scale (VRS) into account. This model evaluates how effectively DMUs transform inputs into outputs, offering a robust measure of efficiency across firms. The following formula illustrates the process:

$$E_{k}=\frac{\Sigma_{j=1}^{J}w_{\mathrm{jk}}y_{\mathrm{jk}}}{\Sigma_{i=1}^{I}z_{\mathrm{ik}}x_{\mathrm{ik}}}$$

The efficiency score *E _k_* reflects the performance of the *k ^th^* Decision-Making Unit (DMU). In this framework:

*y _jk_* stands for the *j ^th^* output produced by the *k ^th^* DMU.

*w _jk_* is the weight assigned to the *j ^th^* output.

*x _ik_* denotes the *i ^th^* input used by the *k ^th^* DMU.

*z _ik_* stands for the weight attributed to the *i ^th^* input.

All input ( *x _ik_*) and output ( *y _jk_*) values are non-negative ( *w _jk_,z _ik_≥*0), and each DMU must have at least one positive input and output for meaningful efficiency analysis.

*3.1.2 Regression model*

The GEE panel regression model was selected after thorough regression diagnostics. Preliminary tests revealed significant within-panel correlation, making traditional models like Ordinary Least Squares (OLS) inefficient due to autocorrelation. The Hausman test showed inappropriateness of the Random Effects model because of correlation between independent variables and individual effects. In this regard, the GEE effectively addresses within-panel correlation, common in longitudinal datasets, by specifying a working correlation matrix ( Liang & Zeger, 1986). An exchangeable correlation structure was adopted, assuming uniform correlation across repeated measures. This approach models relationship between units over time, enabling more precise estimation of populationaveraged effects. Additionally, GEE accommodates various link functions and distributional assumptions, enhancing flexibility and applicability across diverse datasets ( Ghisletta & Spini, 2004; Pan, 2001). The framework is particularly helpful for addressing panel data complexities and improving result interpretability. The specification is as follows:

$$g\left(E\left[Y_{\mathrm{ij}}\right]\right)=\beta_{0}+\beta_{1}X_{\mathrm{ij}1}+\beta_{2}X_{\mathrm{ij}2}\ldots \ldots \ldots \ldots \ldots \ldots +\beta_{k}X_{\mathrm{ijk}}+\epsilon_{\mathrm{ij}}\ldots \ldots \ldots \ldots \left(\mathrm{ii}\right)$$

*Y _ij_* is the dependent variable for the *i ^th^* subject at the *j ^th^* time point. *g* is the link function (identity for continuous outcomes). *X _ijk_* are the independent variables (predictors), including both major effects and interaction terms. *β _k_* are the estimated coefficients. *ϵ _ij_* signifies the random error term. Based on the equation the regression model M1 is created for assessing the impact of MA on WCME.

$$g\left(E\left[{\text{WCME}}_{\mathrm{ij}}\right]\right)=\beta_{0}+\beta_{1}{\mathrm{MA}}_{\mathrm{ij}}+\beta_{2}{\mathrm{ROA}}_{\mathrm{ij}}+\beta_{3}{\mathrm{TBQ}}_{\mathrm{ij}}+\beta_{4}\mathrm{MA}*{\mathrm{TBQ}}_{\mathrm{ij}}+\beta_{5}{\mathrm{LEV}}_{\mathrm{ij}}+\beta_{6}{\text{SIZE}}_{\mathrm{ij}}+\beta_{7}{\mathrm{DBA}}_{\mathrm{ij}}+\beta_{8}{\mathrm{DOC}}_{\mathrm{ij}}+\epsilon_{\mathrm{ij}}\ldots \ldots \ldots \ldots \ldots \ldots \ldots ..M1$$

$$g\left(E\left[{\text{WCME}}_{\mathrm{ij}}\right]\right)=\beta_{0}+\beta_{1}{\mathrm{MA}}_{\mathrm{ij}}+\beta_{2}{\mathrm{ROA}}_{\mathrm{ij}}+\beta_{3}{\mathrm{TBQ}}_{\mathrm{ij}}+\beta_{4}{\text{HQHM}}_{\mathrm{ij}}+\beta_{5}{\mathrm{LEV}}_{\mathrm{ij}}+\beta_{6}{\text{SIZE}}_{\mathrm{ij}}+\beta_{7}{\mathrm{DBA}}_{\mathrm{ij}}+\beta_{8}{\mathrm{DOC}}_{\mathrm{ij}}+\epsilon_{\mathrm{ij}}\ldots \ldots \ldots \ldots \ldots \ldots \ldots ..M2$$

$$g\left(E\left[{\text{WCME}}_{\mathrm{ij}}\right]\right)=\beta_{0}+\beta_{1}{\mathrm{MA}}_{\mathrm{ij}}+\beta_{2}{\mathrm{ROA}}_{\mathrm{ij}}+\beta_{3}{\mathrm{TBQ}}_{\mathrm{ij}}+\beta_{4}{\text{HQLM}}_{\mathrm{ij}}+\beta_{5}{\mathrm{LEV}}_{\mathrm{ij}}+\beta_{6}{\text{SIZE}}_{\mathrm{ij}}+\beta_{7}{\mathrm{DBA}}_{\mathrm{ij}}+\beta_{8}{\mathrm{DOC}}_{\mathrm{ij}}+\epsilon_{\mathrm{ij}}\ldots \ldots \ldots \ldots \ldots \ldots \ldots ..M3$$

$$g\left(E\left[{\text{WCME}}_{\mathrm{ij}}\right]\right)=\beta_{0}+\beta_{1}{\mathrm{MA}}_{\mathrm{ij}}+\beta_{2}{\mathrm{ROA}}_{\mathrm{ij}}+\beta_{3}{\mathrm{TBQ}}_{\mathrm{ij}}+\beta_{4}{\text{LQHM}}_{\mathrm{ij}}+\beta_{5}{\mathrm{LEV}}_{\mathrm{ij}}+\beta_{6}{\text{SIZE}}_{\mathrm{ij}}+\beta_{7}{\mathrm{DBA}}_{\mathrm{ij}}+\beta_{8}{\mathrm{DOC}}_{\mathrm{ij}}+\epsilon_{\mathrm{ij}}\ldots \ldots \ldots \ldots \ldots \ldots \ldots ..M4$$

$$g\left(E\left[{\text{WCME}}_{\mathrm{ij}}\right]\right)=\beta_{0}+\beta_{1}{\mathrm{MA}}_{\mathrm{ij}}+\beta_{2}{\mathrm{ROA}}_{\mathrm{ij}}+\beta_{3}{\mathrm{TBQ}}_{\mathrm{ij}}+\beta_{4}{\text{LQLM}}_{\mathrm{ij}}+\beta_{5}{\mathrm{LEV}}_{\mathrm{ij}}+\beta_{6}{\text{SIZE}}_{\mathrm{ij}}+\beta_{7}{\mathrm{DBA}}_{\mathrm{ij}}+\beta_{8}{\mathrm{DOC}}_{\mathrm{ij}}+\epsilon_{\mathrm{ij}}\ldots \ldots \ldots \ldots \ldots \ldots \ldots ..M5$$

The variance of ${\text{WCME}}_{\mathrm{ij}}$ , conditional on the covariates $\;X_{\mathrm{ij}}^{\prime }$ , is modeled as $\mathrm{Var}\left({\text{WCME}}_{\mathrm{ij}}/X_{\mathrm{ij}}^{\prime }\right)=v\left(Y_{\mathrm{ij}}\right)\phi$ , where v denotes the variance function of $\mu_{\mathrm{ij}}$ and ϕ represents the scale parameter. Given that the dependent variable is continuous, the Gaussian identity link function is employed. The variables were winsorized at the 99 ^th^ percentile to lessen the impact of outliers ( Tukey, 1962; Wilcox, 2017). For robustness check, several diagnostics were conducted, including the Quasi-likelihood in the Independence Model Criterion (QIC), Variance Inflation Factor (VIF), and robust standard errors. A smaller QIC value implies a decent fit, and various correlation structures are compared to identify the one with the smallest QIC ( Pan, 2001). Robust standard errors were used for any potential misspecifications in the correlation structure, ensuring the reliability of the estimates ( Hardin & Hilbe, 2003).

## 4. Analysis

### 4.1 Summary statistics

Table 2 displays the summary statistics. With a mean of 0.204 and a median of 0.151, the WCME variable shows significant variability and a skewed distribution. This reflects diverse working capital management practices across firms. With a comparatively low standard deviation of 0.205 and a mean of 0.59 and median of 0.62, MA shows that managerial ability levels are generally consistent among the organizations. The distribution of ROA seems to be centred, with a mean of 8.57 , median of 8.6, and a low standard deviation. However, the wide range suggests that business profitability varies. TBQ has a right-skewed distribution with a mean of 3.28 and a median of 2.39, driven by firms with consistently high TBQ. With a mean of 0.397, a median of 0.211, and a high standard deviation, LEV shows that various businesses have dynamic financial structure. A limited number of very large firms in the sample are the main cause of the large range in firm size, as displayed by the mean of 17,148.88, median of 4,945.51, and high standard deviation of 37,886.68. Finally, SGR has a mean of 13.15% and a median of 10.49%, accompanied by a standard deviation of 24.65%. This shows significant variability in growth trajectories, highlighting the diverse performance dynamics within the sample.

**Table 2.** Descriptive statistics.

Variable	N	Mean	Median	Std. Dev.	Min	Max
WCME	1500	0.204	0.151	0.181	0.004	1.03
MA	1500	0.593	0.62	0.205	0.119	1.13
ROA	1500	8.565	8.6	4.472	0	31.612
TBQ	1500	3.28	2.39	2.727	0.115	16.915
LEV	1500	0.397	0.211	0.548	-5.231	4.159
SIZE	1500	17148.88	4945.51	37886.68	185.81	398767.26
SGR	1500	13.15	10.49	24.65	-73.21	399.29

Source: Authors Computation.

Table 3 presents the correlation matrix, providing insights into the relationships among key variables. WCME shows relationship with other variables, exhibiting a weak positive correlation with SIZE, implying that large corporations tend to have slightly higher WCME. MA is positively correlated with ROA and TBQ, indicating that managerial ability contributes to profitability and market valuation. Its relationship with LEV suggests that better WCM is more common in smaller, less leveraged firms. ROA demonstrates a moderate positive correlation with TBQ, reflecting that firms with stronger profitability often achieve higher market valuations. TBQ shows a significant negative association with SIZE and LEV, implying that smaller, less leveraged firms are more highly valued. LEV exhibits a positive correlation with SIZE but negative associations with ROA and TBQ, aligns with the view that higher leverage constrains profitability and valuation. SGR is weakly correlated with WCME, ROA, and TBQ, suggesting that sales growth is modestly linked to working capital efficiency, profitability, and market valuation, while showing minimal association with SIZE and LEV. These findings align with the theoretical insight of operational, financial, and managerial factors influencing corporate performance.

**Table 3.** Pairwise correlations.

Variables	WCME	MA	ROA	TBQ	LEV	SIZE	SGR
WCME	1						
MA	0.042 *	1					
ROA	-0.006	0.102 ***	1				
TBQ	-0.099 ***	0.185 ***	0.440 ***	1			
LEV	0.046 *	-0.084 ***	-0.254 ***	-0.306 ***	1		
SIZE	-0.046 *	-0.101 ***	-0.142 ***	-0.167 ***	0.237 ***	1	
SGR	0.060 **	-0.023	0.070 ***	0.103 ***	-0.025	0.002	1

Source: Authors Computation.

*** p<0.01.

** p<0.05.

* p<0.1.

### 4.2 Regression analyses

Table 4 examines the factors influencing WCME through GEE regression. MA appears to have a strong influence on WCME, as showed by its positive and highly significant coefficient. The interaction term between MA and TBQ indicates that the relationship between MA and WCME is moderated by the firm's TBQ, with a stronger negative effect on WCME at higher valuation. TBQ also shows a significant negative effect on WCME, implying that high valued firms seem to have low efficiency in management of working capital. SIZE is negatively associated with WCME, with larger firms showing lower efficiency, which could be due to increased complexity in managing working capitaldue to larger scale of operations. The coefficients for ROA, LEV, DBA, and DOC are not statistically significant. SGR’s significant positive impact on WCME, indicates that higher sales growth causes corporations to manage their working capital more efficiently.

**Table 4.** GEE Regression results (M1).

	Coef.		St.Err.	t-value
*MA*	2.701 ***		0.553	4.89
*ROA*	0.017		0.021	0.82
*TBQ*	-0.111 ***		0.037	-2.98
*MA* * *TBQ*	-2.186 ***		0.443	-4.93
*LEV*	0.243		0.159	1.53
*SIZE*	-.45 ***		0.06	-7.49
*DBA*	-0.012		0.307	-0.04
*DOC*	-0.038		0.172	-0.22
*SGR*	0.01 ***		0.003	3.00
*Constant*	5.127 ***		0.631	8.12
	Mean dependent var	1.662	SD dependent var	3.253
	Number of obs	1500	Chi-square	120.422

Source: Authors Computation.

*** p<0.01.

** p<0.05.

* p<0.1.

**Table 5.** GEE Regression results based on 4 quadrants.

	M2		M3		M4		M5	
	Coef.		Coef.		Coef.		Coef.	
*MA*	0.843 ** (0.406)		0.818 ** (0.406)		0.865 ** (0.405)		0.833 ** (0.406)	
*ROA*	0.0230 (0.021)		0.0210 (0.021)		0.0210 (0.021)		0.0230 (0.021)	
*TBQ*	-0.17 *** (0.035)		-0.17 *** (0.035)		-0.176 *** (0.035)		-0.17 *** (0.035)	
HQHM	-0.2270 (0.182)		--------		--------		--------	
HQLM			-0.41 ** (0.202)		--------		--------	
LQHM	--------		--------		0.651 *** (0.204)		--------	
LQLM	--------		--------		--------		0.0440 (0.18)	
LEV	0.29 * (0.16)		0.291 * (0.16)		0.2350 (0.161)		0.302 * (0.16)	
SIZE	-0.472 *** (0.061)		-0.456 *** (0.061)		-0.47 *** (0.06)		-0.467 *** (0.061)	
SGR	0.01 *** (0.003)		0.01 *** (0.003)		0.01 *** (0.003)		0.01 *** (0.003)	
DBA	-0.1890 (0.309)		-0.1090 (0.308)		-0.1550 (0.307)		-0.1560 (0.308)	
DOC	0.0440 (0.174)		0.0150 (0.173)		0.0150.173		0.0290.174	
Constant	5.423 *** (0.639)		5.344 *** (0.634)		5.264 *** (0.633)		5.311 *** (0.64)	
	Mean dependent var	1.662	Mean dependent var	1.662	Mean dependent var	1.662	Mean dependent var	1.662
	Number of obs	1500	Number of obs	1500	Number of obs	1500	Number of obs	1500
	SD dependent var	3.253	SD dependent var	3.253	SD dependent var	3.253	SD dependent var	3.253
	Chi-square	96.204	Chi-square	98.938	Chi-square	105.392	Chi-square	94.604

Source: Authors Computation.

*** p<0.01.

** p<0.05.

* p<0.1.

The individual quadrants also provide interesting observations as follows:

### 4.3 High Tobin’s Q and High Managerial Ability (HQHM)

For firms categorized under HQHM, MA shows a significant impact on WCME. This highlights the importance of capable management in enhancing efficiency. However, TBQ demonstrates a significant negative effect on WCME, suggesting that high market valuation may be linked to inefficiencies in managing working capital. LEV is marginally significant hinting at a potential impact of debt levels on working capital efficiency. SIZE also negatively affects WCME, while SGR has a marginal positive contribution to efficiency. Interestingly, ROA does not show a significant relationship with WCME, suggesting that profitability may not be a primary driver of working capital efficiency for these firms. Firms in quadrant HQHM benefit from having both skilled managers and high market valuation, but they might face challenges with working capital efficiency if their size increases. Additionally, sales growth appears to positively influence efficiency in WCM.

### 4.4 High Tobin’s Q and Low Managerial Ability (HQLM)

For firms with HQLM, MA continues to positively influence WCME, though the effect is weaker compared to firms in HQHM quadrant. TBQ has a significant negative association with WCME, indicating inefficiencies in WCM despite high market valuation. The HQLM dummy variable shows a significant negative effect, suggesting that differences in managerial ability and market valuation explain variations in WCME within this group. LEV shows similar marginal significance, pointing the effect of debt levels on the efficiency of working capital. SIZE exhibits reduced efficiency in the management of working capital, while SGR remains a positive contributor to WCME. Overall, while managerial ability positively affects WCME in HQLM firms, the influence is less pronounced, with inefficiencies arising from high market valuation, firm size, and debt levels. Thus, for firms in HQLM, there is a moderate positive effect of MA on WCME, . They also face challenges related to market valuation, firm size, and a marginal influence from debt level.

### 4.5 Low Tobin’s Q and High Managerial Ability (LQHM)

For firms with LQHM, MA continues to positively and significantly influence WCME. This reinforces the critical role of skilled management in improving WCME, even when market valuation is low. TBQ remains a significant negative predictor, indicating inefficiencies in working capital management associated with lower market valuation. The HQLM dummy shows a notable adverse impact, suggesting that firms in this quadrant may face additional inefficiencies despite their high MA. LEV is marginally significant, pointing to a possible impact of leverage on WCME. Larger firms (SIZE), continue to struggle with working capital efficiency, while SGR consistently has a significant positive effect, highlighting the influence of sales growth in enhancing working capital management. Overall, LQHM firms rely heavily on MA to improve WCME, but face inefficiencies linked to their lower market valuation and larger size. For firms in LQHM, managerial ability still plays an essential role in improving working capital efficiency, but market valuation and firm size present challenges.

### 4.6 Low Tobin’s Q and Low Managerial Ability (LQLM)

For firms with LQLM, WCME continues to be positively and significantly influenced by MA. This highlights the critical role of management in enhancing WCME, even among firms with both low market valuation and managerial ability. TBQ remains a significant negative predictor, highlighting continual inefficiencies in working capital management linked to lower market valuation. Interestingly, the LQHM dummy, has a positive and significant effect, suggesting that such firms benefit substantially from better managerial capabilities. LEV remains marginally significant, indicating a minor influence of leverage on WCME. Larger firms, face challenges in managing working capital efficiently. Sales growth positively and significantly affects WCME, reinforcing the importance of growth in driving efficiency. Overall, firms in the LQLM quadrant show reliance on managerial ability and sales growth to mitigate inefficiencies associated with their lower valuation and size. Firms with both low market valuation and low managerial ability (LQLM) face significant challenges in managing working capital, but managerial ability plays a crucial role. Even for these businesses, SGR improves efficiency.

### 4.7 Discussions

The regression results show that MA has a favourable effect on WCME, as corroborated in the findings of ( Banerjee & Deb, 2023, 2024) where they suggest that skilled managers have the acumen to navigate both long-term strategic investments and short-term operational efficiency. The observation aligns with the UET, confirming that managerial attributes directly influence organizational outcomes ( Banerjee & Deb, 2023, 2024; Hambrick & Mason, 1984). The significant negative relationship of the interaction term (MA and TBQ) also suggests skilled managers might emphasize strategic, long-term investments rather than operational efficiency in working capital management. It is plausible that managers in such firms, often driven by growth prospects , might prioritize strategic initiatives or long-term investments, potentially leading to a trade-off for WCME ( Banerjee & Deb, 2023; Ujah et al., 2020). This is in line with the current view on the dynamic relationship between long term benefits and working capital management, where efficient WCM can release funds for CAPEX, but a strong focus on CAPEX might also lead to some compromise on WCM efficiency ( Banerjee & Deb, 2023, 2024). The negative association between SIZE and WCME, indicates that larger firms often face increased difficulties in managing working capital efficiently as confirmed previously ( Ujah et al., 2020). This complexity might stem to the need for a more intricate WCM system in larger organizations, which may be hindering optimal efficiency ( Banerjee & Deb, 2023). On the other hand, the positive impact of sales growth on WCME aligns with the understanding that robust sales often translate into improved cash flow, providing managers greater flexibility in managing working capital ( Ujah et al., 2020). This reinforces the prevailing assertion that efficient WCM is a cornerstone for firms aiming to optimize cash flow and fuel sustainable growth ( Banerjee & Deb, 2023). However, the non-significant coefficients for profitability (ROA) and leverage (LEV) deviate from established views. This calls for a further investigation to unravel the specific contextual factors contributing to these results.

The quadrant analysis offers interesting insight into the relationship between MA, TBQ, and WCME. For firms with HQHM, skilled management significantly enhances WCME, yet the high market valuation (TBQ) has a negative effect, potentially reflecting a strategic focus on long-term investments over operational efficiency. These firms also face inefficiencies linked to larger firm, despite marginal benefits from sales growth. In contrast, firms HQLM exhibit a weaker impact of MA on WCME, due to inefficiencies associated with high TBQ and large scale operations. This indicates a limited capacity of low managerial ability to counteract valuationrelated inefficiencies. Firms with LQHM rely heavily on skilled management to drive WCME, even when market valuation is low, but continue to struggle with inefficiencies stemming from size and valuation. SGR plays a consistent positive role among these firms. Lastly, LQLM firms face the most significant challenge, however, still benefit from the incremental contribution of MA to WCME. SGR appears as a key driver of efficiency in these firms as well. Higher sales offer a path to mitigate inefficiencies related to low valuation and firm size.

### 4.8 Theoretical implications

The result of this study has several significant theoretical implications especially when considering the established theories and expanding the understanding of WCM . A positive impact of MA on WCME provides strong empirical support for UET. It suggests that top-level managers' significantly change corporate outcomes. This proves that MA not only affects long-term strategic decisions but also plays a critical role in optimizing short-term operational processes like WCM. Additionally, the negative relationship between MA and TBQ suggests managers may prioritize strategic initiatives over short-term operational efficiency. It indicates a trade-off between longterm investments and WCM efficiency. It calls for a more refined exploration of how high TBQ firms balance CAPEX with WCM needs. The insignificance of profitability (ROA) and leverage (LEV), challenge established beliefs. It signals the requirement of further exploration towards contextual aspects that may explain these unexpected results.

### 4.9 Managerial implications

The results point out the significance of managerial ability in handling working capital efficiently. This should be recognised at top managerial level and corporations should spot the importance of retaining and attracting skilled managers even in operational as well functional levels. It would guarantee optimization of short-term financial decisions and balancing long-term growth strategies. The positive relationship of MA and WCME indicates the importance of investment in talent acquisition, training, and development programs. It would certainly improve managerial wisdom and operational capabilities. Moreover, the study suggests that firms, particularly those with high market valuations, may need to balance the pursuit of strategic growth with the need for efficient working capital management. Managers in such firms must be aware of the potential trade-offs between long term investments and WCM efficiency, ensuring that capital allocation does not come at the expense of operational liquidity. The negative association between firm size and WCME indicates that larger firms face increased complexities in managing working capital effectively, possibly due to more complex operational structures.

## 5. Conclusion

The study focuses on Indian market stressing the importance of skilled managerial administration from an emerging economy perspective. The findings confirm a positive effect of higher MA on WCME. This emphasises how high skill managers look for striking a balance between short term operational efficiency and long-term sustainability. Nevertheless, the interaction term representing MA and TBQ indicates a trade-off in high-growth firms. This hints that these firms are more inclined towards strategic investments in comparison to working capital. From an emerging economy perspective this might be caused due to strategic long term investment objective and higher growth magnitude. The negative association of TBQ with WCME suggest that higher market value does not necessarily ensure higher WCMEshowing a peculiar trend in emerging economy ( Ujah et al., 2020). In terms of size, large corporations have considerable challenges to maintain a desirable WCME. Whereas corporations with better sales growth consistently enhance cash flow and liquidity signifying an improved WCME. The findings align with the UET. It highlights critical effect of top-level managers towards acceptable WCME. This is challenging within the context of India’s diverse industries and firm sizes. Indian firms face liquidity constraints, dynamic sales growth, and frequent regulatory changes. Management of such complexities in these organizations requires customised strategies. Adoption of advanced tools for forecasting, automation, and data-driven WCM systems can enhance and ensure desired efficiency. In general, these findings expand the understanding of managerial ability and its impact on the working capital dimension of Indian firms. It provides a linkage to the existing theories and makes a theoretical linkage with certain fundamental concepts such as RBV, UET and AT. It calls for a refined roadmap from the relevant stakeholders to encourage managerial talent and define actionable strategies that are conducive for consideration of market uncertainty, rapid growth, and regulatory shifts. Future research could be done with other macroeconomic factors that may enhance the findings from a broader perspective. This suggests for a more context-specific approach, where factors such as industry type, firm lifecycle, and macroeconomic conditions could bring more perspective to the observations.

## Data Availability

The data used in the study is obtained from CMIE Prowess software. The software is subscription based, hence the data may not be openly shared. The data used in the study may be obtained from the corresponding author on reasonable requests. Those who wish to subscribe may go to the following website and fill the form for subscription https://prowessiq.cmie.com/. The data that has been used in the study is obtained using the CMIE Prowess database which is a subscription based database. Anyone who wishes to obtain the data may visit the CMIE Prowess database and fill the form, and seek for the subscription by paying the requisite fees. Once they have the access to the database, they can use the database to download the data which we have used in this study.
