# Supplementary material for: Skill and Strategy: How Managerial Ability Drives Working Capital Efficiency
Source: F1000Res. 2025 Mar 28;14:347. [Version 1] doi: 10.12688/f1000research.162551.1 (PMC12013595; doi:10.12688/f1000research.162551.1)
Supplement: Peer review history

# Reviewer report: Reviewer response for version 1

**DOI:** 10.5256/f1000research.178769.r374110

**Published:** 2025-04-16

**Authors:** Ram Pratap Sinha

**Affiliations:** 1 Government College of Engineering and Leather Technology, Kolkata, West Bengal, India

Resource management is crucial for firms to achieve operational and financial objectives. Managers optimise allocating overall resources and short-term capital to drive profitability and long-term sustainability. Given the above, the manuscript considers the linkage between managerial ability and working capital efficiency. The paper is well presented and provides valuable information about the relationship in Indian context.

Is the work clearly and accurately presented and does it cite the current literature?

Yes

If applicable, is the statistical analysis and its interpretation appropriate?

Yes

Are all the source data underlying the results available to ensure full reproducibility?

Yes

Is the study design appropriate and is the work technically sound?

Yes

Are the conclusions drawn adequately supported by the results?

Yes

Are sufficient details of methods and analysis provided to allow replication by others?

Yes

Reviewer Expertise:

Data Envelopment Analysis, Stochastic Frontier Analysis, Managerial Ability.

I confirm that I have read this submission and believe that I have an appropriate level of expertise to confirm that it is of an acceptable scientific standard.

# Reviewer report: Reviewer response for version 1

**DOI:** 10.5256/f1000research.178769.r374103

**Published:** 2025-04-08

**Authors:** B. G. Guruprasad B. G.

**Affiliations:** 1 Surana Evening College, Bengaluru, Karnataka, India

Summary of the Article

 The study investigates the impact of Managerial Ability on Working Capital Management Efficiency, emphasizing how skilled management influences working capital practices. Using a balanced panel dataset of 150 listed firms from the NSE of India (2014–2023), the research employs Data Envelopment Analysis and Generalized Estimating Equations regression models to assess the relationship between Managerial Ability, Tobin’s Q, and Working Capital Management Efficiency. The findings suggest that higher managerial ability enhances Working Capital Management Efficiency, particularly in firms with lower market valuations, aligning with the Upper Echelon Theory that managerial attributes are key to organizational efficiency. The study offers significant contributions to corporate finance literature by integrating Managerial Ability and Working Capital Management Efficiency in an emerging market context.

 Strengths of the Article

 •    Robust Methodological Approach – The integration of DEA and GEE models provides a rigorous analytical framework, ensuring precise measurement of efficiency and managerial ability.

 •    Empirical Contribution to Indian Corporate Finance – The study fills a gap in the literature on working capital efficiency and managerial effectiveness in emerging markets by focusing on Indian listed firms.

 •    Multi-Dimensional Assessment of WCME – The study incorporates inventory, accounts receivable, accounts payable, cost of goods sold, net revenue, and net income, making the efficiency measure more comprehensive.

 •    Alignment with Established Theories – The research connects findings with Upper Echelon Theory, Resource-Based View  and Agency Theory, strengthening its theoretical foundation.

 •    Managerial Relevance – The results offer practical insights for corporate leaders, suggesting that firms should enhance managerial skill development to improve WCME and achieve long-term sustainability.

 Major Points for Improvement 

 1.    Integration of Cross-Disciplinary Perspectives – While the study focuses on skill and strategy in a particular domain, expanding it to include insights from cognitive psychology, behavioural economics, or artificial intelligence-driven decision-making could offer a broader theoretical foundation.

 Minor Points for Improvement

 1.    Expansion to Unlisted and SME Firms – The study focuses on publicly listed firms from the NSE. Exploring how MA affects WCME in unlisted companies and small and medium enterprises (SMEs) could provide a broader perspective.

 2.    Exploring Cultural and Regional Variations –A global perspective, analyzing differences in strategic approaches across diverse cultural settings, would enhance its universality.

 3.    Updating References – Incorporating the latest research (2023–2025) would ensure the study remains current.

 The study makes a significant contribution to the literature by examining the relationship between managerial ability and working capital efficiency using advanced methodological tools. While the suggested improvements extend beyond the study’s scope, they highlight future research directions. The article, in its current form, is suitable for indexing.

Is the work clearly and accurately presented and does it cite the current literature?

Yes

If applicable, is the statistical analysis and its interpretation appropriate?

Yes

Are all the source data underlying the results available to ensure full reproducibility?

Yes

Is the study design appropriate and is the work technically sound?

Yes

Are the conclusions drawn adequately supported by the results?

Yes

Are sufficient details of methods and analysis provided to allow replication by others?

Yes

Reviewer Expertise:

Finance, Accounting & Taxation

I confirm that I have read this submission and believe that I have an appropriate level of expertise to confirm that it is of an acceptable scientific standard.

# Reviewer report: Reviewer response for version 1

**DOI:** 10.5256/f1000research.178769.r374111

**Published:** 2025-04-03

**Authors:** Qian Long Kweh

**Affiliations:** 1 Canadian University Dubai, Dubai, United Arab Emirates

This study offers a relevant and well-executed analysis of how managerial ability affects working capital management efficiency (WCME) in large Indian firms. The authors use Data Envelopment Analysis (DEA) to measure efficiency (managerial ability - MA) and Generalized Estimating Equations (GEE) for regression modeling. These approaches are methodologically appropriate. However, several important revisions are needed to meet the publication standards.

 The theoretical framework is currently too broad and should be narrowed to focus on one or two main theories, such as UET and RBV, to improve coherence. Moreover, the writing should be more concise and avoid vague or technical language that could confuse readers. Updating references with more recent studies from top-tier journals would also strengthen the paper’s relevance and credibility.

 The most serious issue is the lack of control for endogeneity, which raises doubts about whether managerial ability truly causes improvements in WCME.

 First, DEA assumes a deterministic production frontier, ignoring measurement error and noise. This is problematic, especially when applied to a decade of financial data across heterogeneous firms. The lack of sensitivity analysis or alternative models like Stochastic Frontier Analysis (SFA) limits confidence in the efficiency estimates. Second, while GEE accounts for within-panel correlation, it fails to address endogeneity. The link between MA and WCME is likely bi-directional, whereby better WCME may attract capable managers, and vice versa. GEE offers population-averaged estimates, not causal inference, unless strict assumptions are met. No IVs, lagged variables, or dynamic panel methods (e.g., System GMM) are employed to mitigate this. Third, MA is measured using a two-step DEA and regression approach per Demerjian et al. (2012), but the paper omits key details about the first-stage regression. This raises doubts about whether firm-level heterogeneity was properly controlled.

 Last but not least, the manuscript would benefit from clearer visual representations of the results, such as charts showing how the impact of managerial ability changes across different firm types.

Is the work clearly and accurately presented and does it cite the current literature?

Partly

If applicable, is the statistical analysis and its interpretation appropriate?

Partly

Are all the source data underlying the results available to ensure full reproducibility?

Partly

Is the study design appropriate and is the work technically sound?

Partly

Are the conclusions drawn adequately supported by the results?

Partly

Are sufficient details of methods and analysis provided to allow replication by others?

Partly

Reviewer Expertise:

Performance Management, Data Envelopment Analysis, Financial Accounting and Reporting.

I confirm that I have read this submission and believe that I have an appropriate level of expertise to confirm that it is of an acceptable scientific standard, however I have significant reservations, as outlined above.
